# Structural and functional microbial diversity of sandy soil under cropland and grassland

**DOI:** 10.7717/peerj.9501

**Published:** 2020-09-02

**Authors:** Magdalena Frąc, Jerzy Lipiec, Bogusław Usowicz, Karolina Oszust, Małgorzata Brzezińska

**Affiliations:** Institute of Agrophysics, Polish Academy of Sciences, Lublin, Poland

**Keywords:** Land use, Fungal community, Functional diversity, Soil enzymes

## Abstract

**Background:**

Land use change significantly alters soil organic carbon content and the microbial community. Therefore, in the present study, the effect of changing cropland to grassland on structural and functional soil microbial diversity was evaluated. The specific aims were (i) to identify the most prominent members of the fungal communities and their relevant ecological guild groups; (ii) to assess changes in the diversity of ammonia-oxidizing archaea; (iii) to determine the relationships between microbial diversity and selected physical and chemical properties.

**Methods:**

We investigated microbial diversity and activity indicators, bulk density and the water-holding capacity of sandy soil under both cropland and 25-year-old grassland (formerly cropland) in Trzebieszów, in the Podlasie Region, Poland. Microbial diversity was assessed by: the relative abundance of ammonia-oxidizing archaea, fungal community composition and functional diversity. Microbial activity was assessed by soil enzyme (dehydrogenase, β-glucosidase) and respiration tests.

**Results:**

It was shown that compared to cropland, grassland has a higher soil organic carbon content, microbial biomass, basal respiration, rate of enzyme activity, richness and diversity of the microbial community, water holding capacity and the structure of the fungal and ammonia-oxidizing archaea communities was also altered. The implications of these results for soil quality and soil health are also discussed. The results suggest that grassland can have a significant phytosanitary capacity with regard to ecosystem services, due to the prominent presence of beneficial and antagonistic microbes. Moreover, the results also suggest that grassland use may improve the status of soil organic carbon and nitrogen dynamics, thereby increasing the relative abundance of fungi and ammonia-oxidizing archaea.

## Introduction

Soil is the fundamental resource of an agricultural ecosystem (Wang et al., 2016). Different land uses significantly alter the soil organic carbon stock and structure, which in turn influences soil properties, functions and the composition of the soil microbiome (Delelegn et al., 2017; Szoboszlay et al., 2017). Land use and changes in the use of agricultural land are largely influenced by market prices, technology and policy that makes one type of land use more cost-effective compared to another (Claassen, Carriazo & Ueda, 2010). During the last few decades, the conversion of grassland and pasture to cultivated croplands has occurred in many regions of the world (Claassen, Carriazo & Ueda, 2010; Wright & Wimberly, 2013). This process has been enhanced by the increased availability of more productive crop varieties (Claassen, Carriazo & Ueda, 2010), and the move from conventional to no-till production using fewer field machinery operations and more recently due to crop-based biofuel feedstock production (Lal, 2005; Wright & Wimberly, 2013; Qin et al., 2016).

This shift has been widely observed on highly productive fine textured soils (Claassen, Carriazo & Ueda, 2010). From the soil quality point of view an important concern associated with land use change is the change in soil organic carbon (SOC) (Vaccari et al., 2012). Many studies have shown that converting land from less intensive grasslands to more intensive croplands has resulted in a decrease in soil carbon stocks (Claassen, Carriazo & Ueda, 2010; Deng et al., 2016). A recent literature review of Qin et al. (2016) revealed that regardless of soil depth and study duration, the conversion from grassland to corn production has caused a substantial decline in carbon stocks of between 9% and 35%. This conversion may lead to a further increase in the intensity of soil erosion, nutrient runoff and leaching and thereby cause environmental damage (Claassen, Carriazo & Ueda, 2010; Bakker et al., 2005).

With regard to low productivity coarse textured soils, the conversion of grassland to croplands is much less common than for finely textured soils (Claassen, Carriazo & Ueda, 2010; Qin et al., 2016; Gosling, Van der Gast & Bending, 2017). However, the conversion of croplands to grasslands using such soil is considered to be an opportunity to increase its carbon sequestration potential and increase initially low SOC. It has been shown that changing croplands to grassland may lead to the sequestration of 0.3–1.7 t C ha^−1^ each year (Qin et al., 2016) and enhance the contribution of aromatic compounds to SOC (Gosling, Van der Gast & Bending, 2017). However, it has been established that a relatively long period of time is required to reach a new SOC equilibrium.

Most research concerning land use change has focused on soil chemical composition and soil organic carbon stocks (Qin et al., 2016; Claassen, Carriazo & Ueda, 2010) as well as microbial biomass carbon (Maková et al., 2011; Gosling, Van der Gast & Bending, 2017) and the structure of the bacterial community (Kaiser et al., 2016; Millard & Singh, 2010). However, to date there has been a lack of research in the areas of functional diversity, the fungal microbiome and biochemistry even though they affect many soil processes and functions. Soil biodiversity represents a complex underground world involving a wide range of organisms, from archaea to fungi, that interact with each other and affect the functioning of the soil ecosystem (Schmidt et al., 2013).

Soil nitrification, as a step in nitrogen cycling, plays an important role in the loss of ammonium connected with atmospheric and water pollution by nitrous oxide and nitrate, respectively (Prosser & Nicol, 2012). The nitrate, which impacts directly on plant growth rates, can be produced in heterotrophic or autotrophic pathways. Ammonia-oxidizing archaea (AOA) are the key autotrophic contributors to ammonia oxidation, and their comparative input into this process is one of the most relevant with reference to the nitrogen cycle in soil (Leininger et al., 2006). They predominate among the ammonia-oxidizing prokaryotes in soils (Leininger et al., 2006) including those under grasslands (Clark et al., 2020) and are favoured by low soil pH (Prosser et al., 2020). In addition, the AOA group of microbes show a high ammonia affinity and play an important role in nitrification (Beeckman, Motte & Beeckman, 2018; Prosser & Nicol, 2012). However, heterotrophic nitrification, in which fungi converts organic N to nitrate may also be substantial (Wardle et al., 2004; Cookson et al., 2006). Broad changes in the structure of the soil fungal community and AOA diversity, may directly influence soil properties and have a direct impact on ecosystem productivity. Moreover, one of the main factors that determines the rates of nitrification in agricultural soils includes the populations of nitrifiers and competitors (Norton & Ouyang, 2019). In general ammonia oxidizing archaea predominate acidic soils, while soils with ~7.0 and higher pH values are associated with ammonia oxidizing bacteria (AOB) rather than AOA (Che et al., 2015). Therefore, we focused on the diversity of the AOA group of microbes and also on the less well known fungal communities that could be co-competitive microorganisms for nitrifiers in sandy acidic soils. Moreover, the determination of fungal biodiversity and structure is very useful in the assessment of its functionality and role in soil health and quality, this process is effective due to the use of high throughput sequencing approaches (Frąc et al., 2018). Therefore, in the research presented, we examined the effect of land use change from croplands to grassland, 25 years after conversion, with regard to the relative abundance of AOA and the structure of the fungal community using modern molecular methods in correlation with other conventional soil quality indicators of low fertility sandy soil. A better understanding of fungal composition, its relevant ecological guild groups, AOA diversity and relationships with other soil properties in grassland and cropland provides a wide range of knowledge concerning microbial community changes important in the management of land use, which should be highlighted as a particularly noteworthy novelty of this research. The effect of changing cropland to grassland on structural and functional soil microbial diversity was evaluated in this study. The specific aims were: (i) to identify the most prominent members of the fungal communities and their relevant ecological guild groups; (ii) to assess changes in the diversity of ammonia-oxidizing archaea; (iii) to determine the relationships between microbial diversity and selected physical and chemical properties of the soil.

## Materials and Methods

### Site description and soil sampling

The experimental site was located in Trzebieszów in the Podlasie Region of Poland (51°59′09.8″N, 22°33′57.5″E) and included sandy acidic soil under cropland and 25-years-old grassland (converted cropland). The area of each neighbouring cropland and grasslands field was 0.5 ha. The crop rotation regime of the cropland included potato–oats–barley–triticale. The grassland was composed mainly of the meadow fescue (*Festuca pratensis* Huds.). The access authorization to the fields were given verbally by farmer Marek Lasocki, owner of the agricultural farm and these fields. The soil is Podzol (WRB IUSS Working Group, 2015) formed from a sandy material of glacial origin and considered to have a low productivity value. It contains 86% sand, 12% silt, 2% clay and it is acidic. The area has a continental climate with a mean annual temperature of 7.3 °C and annual precipitation of 565 mm. The altitude of the study site is approximately 150 m a.s.l.

The soil quality indicators were evaluated with reference to the two above-mentioned land uses: cropland (CL) and grassland (GL) from three plots (I–III) established in each field as three biological replicates. The moisture contents of the plots ranged from 8.45% to 24.06%. Soil samples (0–10 cm depth) were collected in 2016 in separate triplicates for each land use type. In order to obtain a representative sample for each land use type, from each of the three plots soil samples were collected from 21 individual points, pooled together in plastic bags, placed on ice and transported to the laboratory. Each soil sample was mixed to homogenize it, and then passed through a 2-mm sieve and analysed.

### Microbiological analyses

To determine the basal respiration rate (BR), 10 g soil samples were incubated at 20 °C in 120 cm^3^ glass vials, tightly sealed with rubber stoppers and aluminum caps. Headspace CO_2_ concentration was measured after 4 h of incubation by collecting a 200 µl gas sample and analysing it using gas chromatography. The BR was calculated based on the amount of CO_2_ released by soil, and expressed in mg CO_2_–C per kilogram dry mass soil per hour. The soil microbial biomass C content (MBC) was assayed using the substrate induced respiration (SIR) method based on the initial respiratory response of the microbial population (CO_2_ release) to amendment with an excess of glucose as an easily available carbon and energy source (Anderson, 2003). Soil samples (10 g dry mass) were transferred into 60 cm^3^ glass vials and amended with five ml of 1% glucose solution (corresponding to an amendment of five mg glucose per gram of dry soil). Soil slurries were incubated while being shaken at 25 °C using a water bath. After 4 h of incubation, the CO_2_ evolved was determined using gas chromatography. The microbial biomass was calculated using the formula: MBC (mg C g^−1^) = 50.4 × (cm^3^ CO_2_ g^−1^ h^−1^) (Šimek & Kalčik, 1998). The concentrations of CO_2_ in the headspace were measured with a GC-14A gas chromatograph (Shimadzu, Kyoto, Japan) equipped with a thermal conductivity detector, TCD (Lipiec et al., 2015). All of the measurements were performed in triplicate. The metabolic potential of the soil bacterial community was evaluated using Biolog EcoPlates™ (Biolog Inc., Hayward, CA, USA) with 31 carbon sources (Insam, 1997), according to the procedure described by Oszust et al. (2014). On the basis of data obtained as the mean value from 216 incubation hours, the Richness (R) index was calculated following the procedure of Garland & Mills (1991) by determining the number of oxidized carbon substrates using an OD ≥ 0.25 as the threshold for a positive response.

Dehydrogenase activity (DA) was determined using the Thalmann method (Thalmann, 1968), modified by Alef (1995) with 2,3,5-triphenyl-tetrazolium chloride (TTC) as a substrate. β-glucosidase activity (BA) was determined according to the Eivazi & Tabatabai method (Eivazi & Tabatabai, 1988) after soil incubation with a substrate: *p*-nitrophenyl-β-d-glucoside (PNG). Enzyme assays were performed in biological triplicates.

Total genomic DNA was extracted from all soil samples using a FastDNA®SPIN Kit for Faeces (MP Biomedicals, Irvine, CA, USA) according to the manufacturer’s protocol. The amount of DNA was determined spectrophotometrically using NanoDrop (Thermo Scientific, Waltham, MA, USA) at a wavelength of 260 nm.

The relative abundance of ammonia-oxidizing archaea (AOA) under both types of land use was characterized by terminal restriction fragment length polymorphism (t-RFLP) analysis of the amoA gene. PCR products of the ammonia monooxygenase α-subunit (amoA) gene were obtained with the use of the following primer pairs: CrenamoA23f: 6-carboxyfluorescein-FAM 5′-ATGGTCTGGCTWAGACG-3′ (Tourna et al., 2008) and amo643R: 5′-TCCCACTTWGACCARGCGGCCATCCA-3′ (Treusch et al., 2005). Then the amplicons were digested using the AluI restriction enzyme. A detailed description of the applied procedure has been published by Oszust et al. (2014). The results of particular samples were collected in blue channel of 3130 xl ABI capillary sequencer, because the forward primers were labelled with FAM dye, while orange channel detected peaks of the standard of DNA fragment size (GS-600LIZ, ABI). Therefore, the results from blue channel, including size, height and area of the peaks, were exported for the next calculations. Then obtained restriction fragments were sorted according to fragment size, results with the size lower than 50 bp were removed, the absolute values of the difference in length of adjacent fragments were calculated, if the difference in the lengths of the relevant fragments was less than one bp the peaks were pooled within one sample. Finally, the sum of all peaks area within one sample and then the percentage of peak area for particular samples were calculated. Because some of peaks can represent a noise of the sequencer, it is recommended to establish a threshold to remove these artefacts, usually for peaks constituting less than 1–5% of the peaks area sum. In this study the threshold was established for peaks that accounted for less 1%. The relative quantification of individual terminal restriction fragments within the PCR products reflects changes in community structure or the relative abundance of AOA. Based on in silico analysis the terminal restriction fragments (t-RFs) were assigned to the *amo*A_AOA Feifei-Liu reference database from the FunGene functional gene pipeline and repository (Fish et al., 2013) and using TRiFLe software (Junier, Junier & Witzel, 2008).

An analysis of the fungal community structure was performed on the basis of the ITS1 region using a primer set ITS1FI2/5.8S (5′-GAACCWGCGGARGGATCA-3′; 5′-CGCTGCGTTCTTCATCG-3′) (Schmidt et al., 2013; Vilgalys Mycology Lab, 1992) using next generation sequencing (NGS) within the Illumina MiSeq Platform in Genomed S.A. (Warsaw, Poland). PCR amplification was carried out in a Q5 Hot Start High-Fidelity 2X Master Mix according to the manufacturers’ protocol. The DNA library was sequenced using the platform of Illumina MiSeq and pair-end technology, 2 × 250 bp with the v2 Illumina kit.

### Physical and chemical analyses

The soil water content and bulk density were determined gravimetrically using 100 cm^3^ cores and the water holding capacity was assumed to be the water content at matric potential 158 hPa (pF 2.2). Soil pH was measured potentiometrically. Soil organic carbon (SOC) was assessed using the modified Tiurin method (Oleszczuk, 2008). The above measurements were performed in three biological replicates-plots of fields (I–III) in four technical replicates for each plot.

### Data analysis

In order to determine if the correlations between the individual soil variables differ as a function of land use types, we analysed different scenarios of correlations, including an analysis of samples only from cropland, only from grassland and all samples from both land use types. Differences in the tested soil properties were determined under scenarios in which both tested soil types were used and by using samples only from cropland or only from grassland, they were explored further using the Principal Component Analysis (PCA). On the basis of cluster analysis from the normalized data of soil properties, a dendrogram was prepared with scaled similarity (%) on the axis (Ward’s method and the Unweighted Pair Group Method with Arithmetic Mean—UPGMA) and the boundary marked according to Sneath’s criteria. A heat map, which shows the relationship between cropland and grassland soils was generated based on the normalized data of the soil properties by the automatic formula including: standardized value = (raw value of variable—mean of variable)/standard deviation of variable. An analysis of variance (ANOVA) with a post-hoc Tukey’s (HSD) test was used to determine the differences between the tested types of land uses (cropland and grassland). The analyses were performed using Statistica v.13.1 software.

MiSeq Reporter (MSR) v2.6. software was used to elaborate the data on a preliminary basis and the Quantitative Insights into Microbial Ecology (QIIME) tool was used to process the raw sequence reads (Caporaso et al., 2010). The taxonomical classification of OTUs was performed using a Basic Local Alignment Search (BLAST) against the UNITE database. The Illumina sequencing data were uploaded in the NCBI Sequence Read Archive database with the accession number SRP131723. The results of this classification were subsequently analysed and visualized using KRONA software (Ondov, Bergman & Phillippy, 2011). The FUNGuild online application was used to assign functional information to OTUs in high-throughput sequencing datasets by its assignment to an ecological guild (Nguyen et al., 2016).

## Results

### Individual soil variable correlations

**Figure 1:**
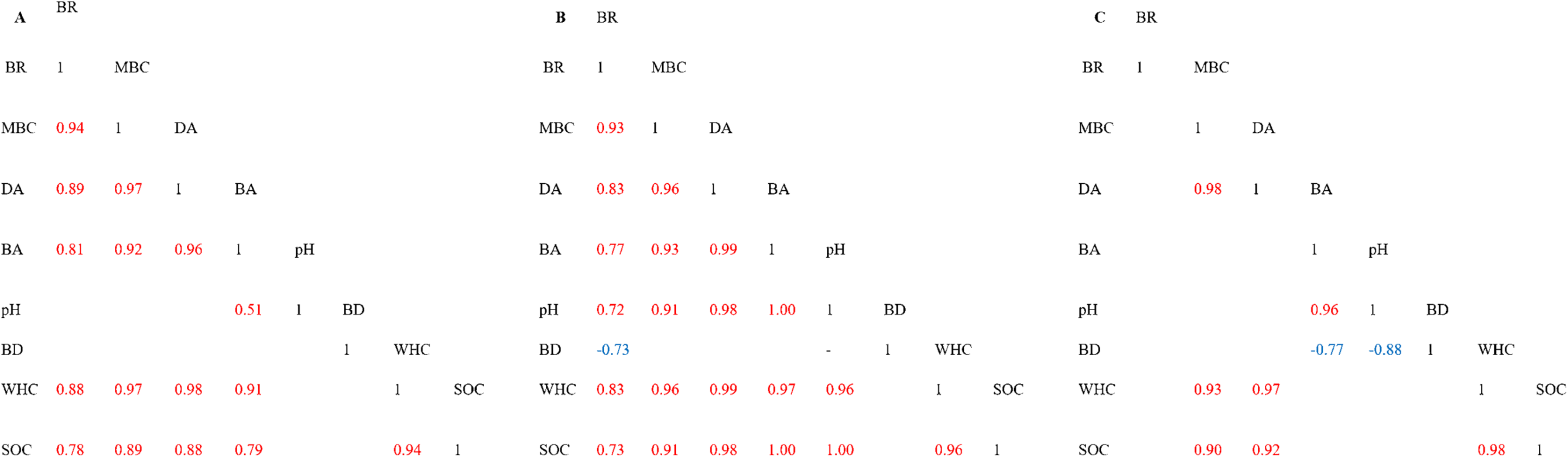
Correlation plot of soil properties within the tested plots of cropland and grassland. (A) All samples within the tested plots of grassland and cropland; (B) only samples originating from grassland; (C) only samples originating from cropland. Explanations: BR, Basal respiration; MBC, Microbial biomass; DA, Dehydrogenase activity; BA, β-glucosidase activity; BD, Bulk density; WHC, Water holding capacity; SOC, Soil organic carbon.

The correlation plots between soil variables indicated that the use of samples from different origins within the experiment may be influential (Fig. 1). The general trend was that for the scenarios using all samples and only those from grassland, the correlations were positive and relatively close. However, for the cropland samples, we observed both positive and negative close correlations. In particular, in all of the scenarios tested, close positive correlations were found between soil variables MBC, DA and WHC and between these and SOC (Figs. 1A–1C). BD was not correlated with any variables in testing scenarios involving all of the samples. However, the use of samples from both grassland and cropland produced negative and moderately close correlations between BD with BR in GL (Fig. 1B) and BD with BA and pH in CL (Fig. 1C), respectively. The pH was positively correlated with BA across all sampling origin testing scenarios (Fig. 1A), with MBC, DA, BA, WHC and SOC in the samples collected from grassland (Fig. 1B) and with BA in cropland (Fig. 1C). Moreover, it is noteworthy that within BR the correlations were substantial and positive between almost all soil variables in the testing scenario using all soil samples (Fig. 1A). All variables were correlated with BR in grassland (Fig. 1B) and no correlations with BR were observed in cropland (Fig. 1C). The SOC was closely related to almost all soil variables across all of the different testing scenarios (Figs. 1A–1C).

**Figure 2:**
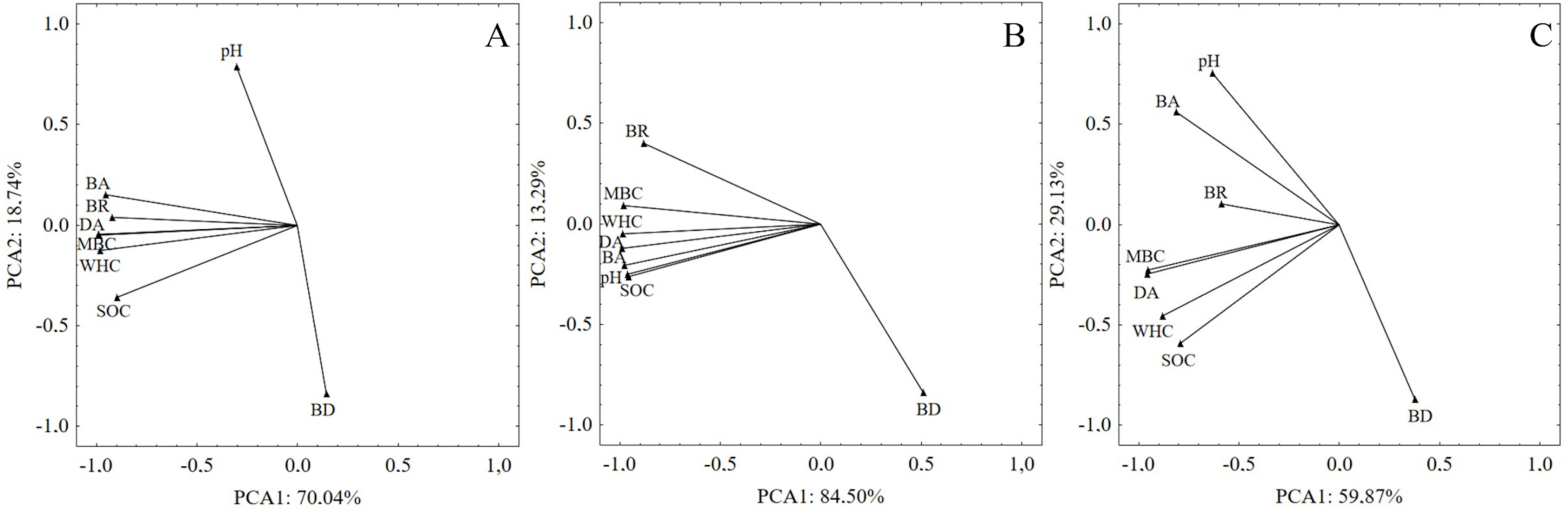
Principal Components Analysis (PCA) of soil properties within the tested land use types: cropland and grassland. (A) PCA by using all samples within grassland and cropland; (B) PCA by using samples originating from grassland; (C) PCA by using samples originating from cropland.

A principal component analysis (PCA) based on soil variables was performed to determine the general differences between land use type (grassland and cropland). A PCA analysis generated two components. All of the variables for all of the tested scenarios are presented graphically in Fig. 2. The first and second principal components (PCA1 and PCA2) explained 88.78%, 97.79% and 89.00% of the total variability of the data set for both land use types, grassland and cropland, respectively. Across all testing scenarios the overall trend was the grouping of selected variables, as follows: BA, DA, MBC, WHC, BR, SOC in all tested samples, MBC, WHC, DA, BA, pH, SOC in grassland and MBC and DA in cropland (Figs. 2A–2C). This general pattern was mainly driven by MBC and DA, which had the highest values in the testing scenario concerning all samples. It was found that lower BR values occurred in cropland (Fig. 2C) compared to grassland (Fig. 2B). However, when all of the samples from grassland and cropland were considered (Fig. 2A) BR was found to have higher values than those found in cropland.

**Figure 3:**
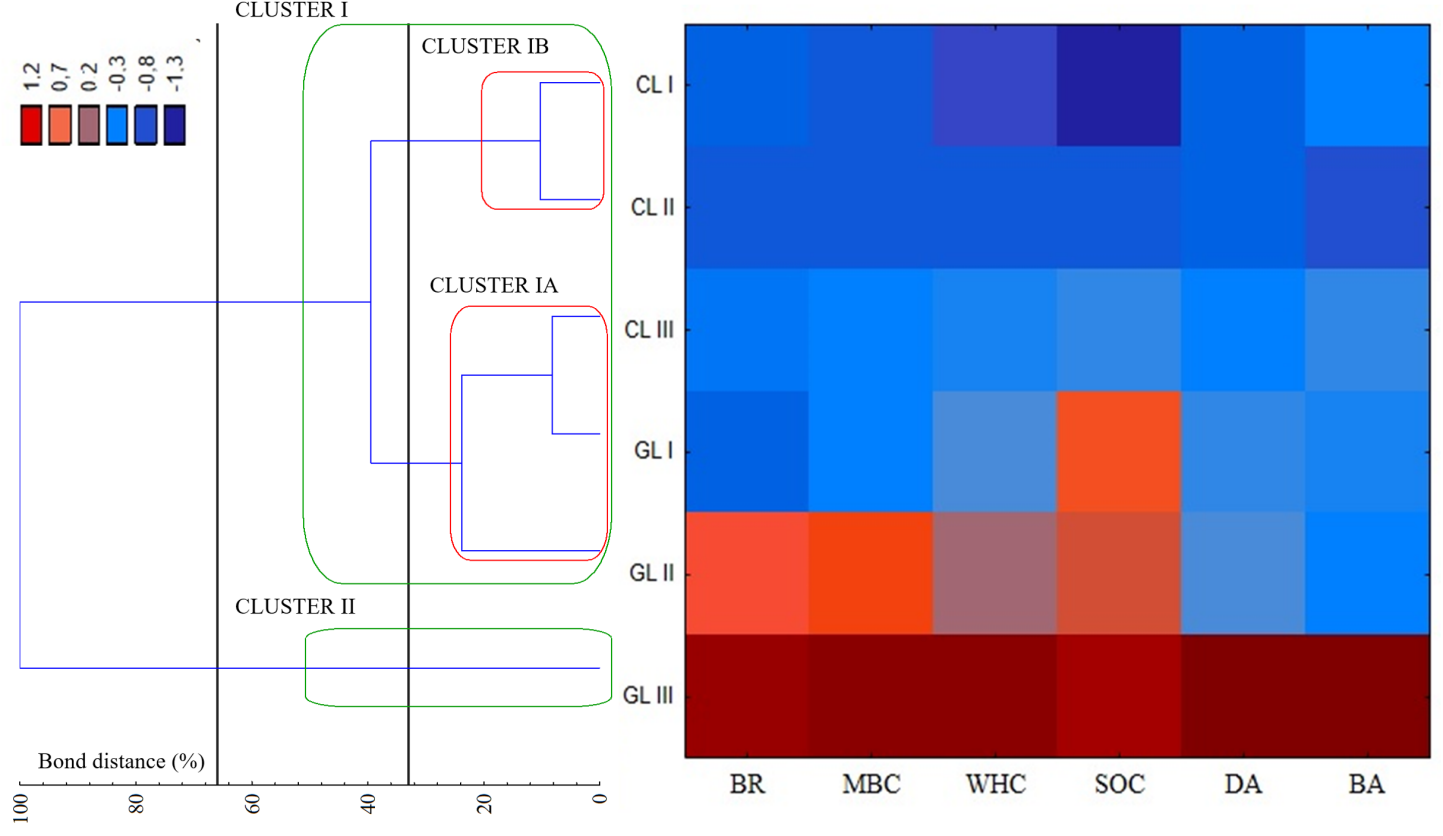
Dendrogram and heat map showing the distribution of plots associated with soil quality indicators of cropland and grassland. Grouping was conducted according to the stringent Sneath criterion (33%) and the less restrictive criterion (66%), respectively. GL, Grassland, CL, Cropland, I–III, Numbers of plots, BR, Basal respiration; MBC, Microbial biomass; DA, Dehydrogenase activity; BA, β-glucosidase activity; BD, Bulk density; WHC, Water holding capacity; SOC, Soil organic carbon.

Figure 3 shows the results of a cluster analysis of the experimental objects based on the tested soil properties. The results indicated that they can be divided into two groups according to the activity and values of the tested parameters. The first group includes all cropland samples and two samples from grassland. Furthermore, inside the first cluster, we observed two separate groups with cropland (Cluster IB) and grassland samples plus one cropland sample (Cluster IA). Cluster IA consisted of samples with moderate values of the tested properties, while cluster IB had the lowest values for the parameters tested (Fig. 3). The second cluster had only one sample from grassland, with the highest values of all tested microbiological and physical properties (Fig. 3). Results presented on heat map concerning the groups of soil samples were normalized in Statistica™ software, to obtain standardized values of soil properties in grassland and cropland. The results indicated that all of the tested parameters had higher values in grassland than in cropland soil samples. In addition, the highest values were noted for SOC in grassland. On the basis of the heat map presented, three cropland samples are grouped together as were the soil samples from grassland, but separated from cropland (Fig. 3). It is noteworthy, that GL III was characterized by the highest values of all of the parameters tested, this may be connected with the hydrological conditions of the soil (samples from this plot were characterized by the highest water content ~24%).

### Basal respiration, microbial biomass content and the richness index

**Figure 4:**
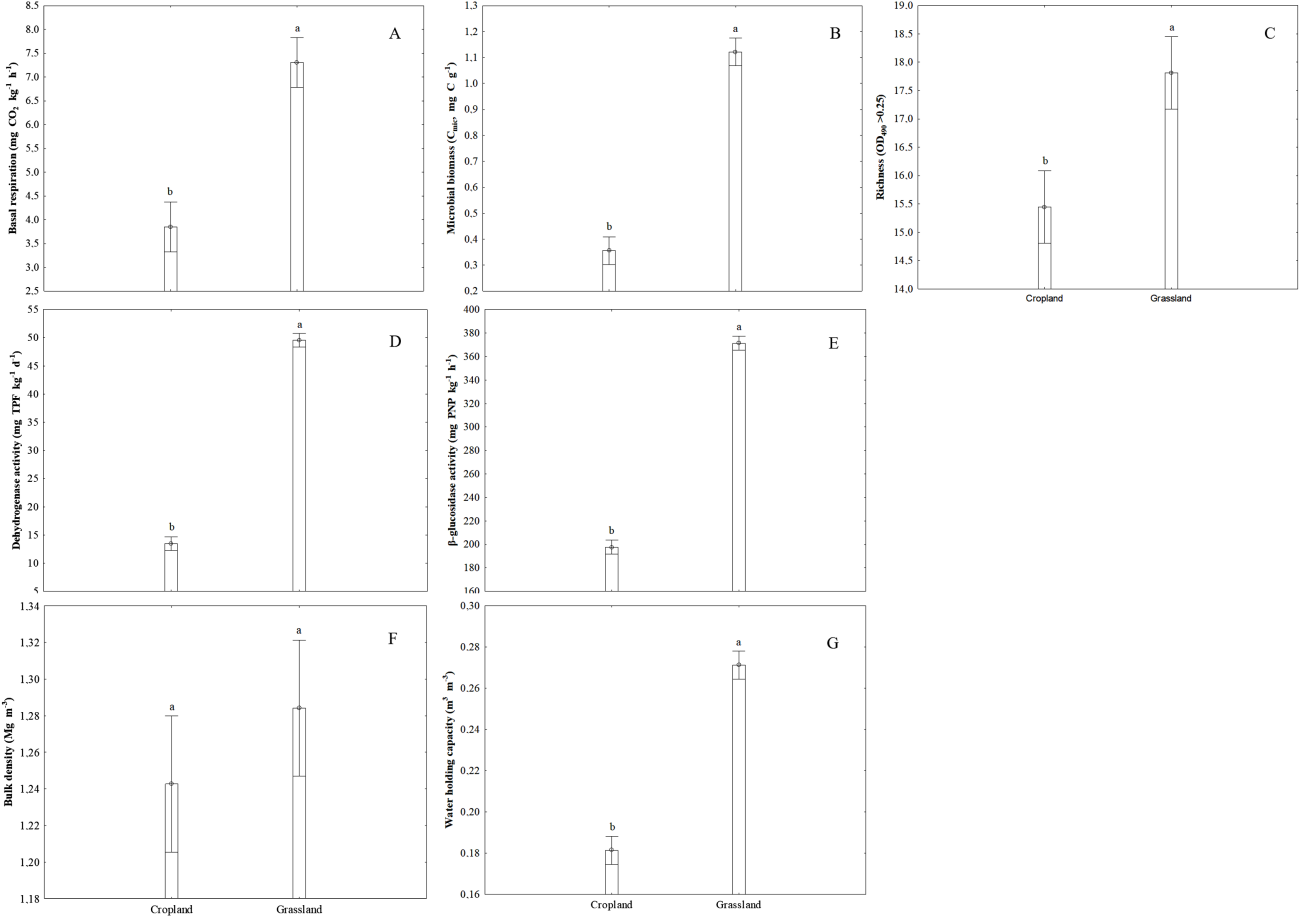
Microbiological, enzymatic and physical properties in the soils of two different land use types: cropland and grassland. (A) Basal respiration, (B) microbial biomass, (C) richness index, (D) dehydrogenase activity, (E) β-glucosidase activity, (F) bulk density, (G) water holding capacity. Vertical bars denote 0.95 confidence intervals. Different letters indicate significant differences between the tested land use types (*P* < 0.05).

Basal respiration and microbial biomass content were substantially influenced by agricultural land use type (Figs. 4A and 4B), indicating that CO_2_ release significantly increased in grassland compared to cropland soil. The highest BR values were noted in plot III of the grassland soil. The mean CO_2_ concentrations were stable in all plots of cropland soil and significantly lower than those of the grassland soil (Fig. 4A). The same trend was observed for microbial biomass content (Fig. 4B). In general, the substrate richness index (R) was significantly higher in grassland compared to cropland, with the exception of plot III, where no significant differences were observed (Fig. 4C).

### Enzymatic activity

The results revealed significant differences in enzymatic activity (DA, BG) between the tested grassland and cropland soils (Figs. 4D and 4E). In general, both tested enzymes (dehydrogenase and β-glucosidase) had significantly higher activities in grassland soils collected from all plots (I–III) than in cropland soils. The highest activity of the tested enzymes was noted in grassland soils collected from plot III. The lowest values were observed in cropland soils from plot I for DA (Fig. 4D) and plot II for BA (Fig. 4E).

### Physical properties of soils

Bulk density and water holding capacity differed significantly (*P* < 0.05) between the types of agricultural use of the soil (Figs. 4F and 4G). Comparing plot I of cropland and grassland, the bulk density was higher (1.22 Mg m^−3^) in the cropland than in the grassland (1.37 Mg m^−3^). In the other plots (II and III) significant differences were not observed (Fig. 4F). The water holding capacity was significantly higher in all plots of grassland as opposed to cropland soils (Fig. 4G).

### Ammonia-oxidizing archaea community

**Figure 5:**
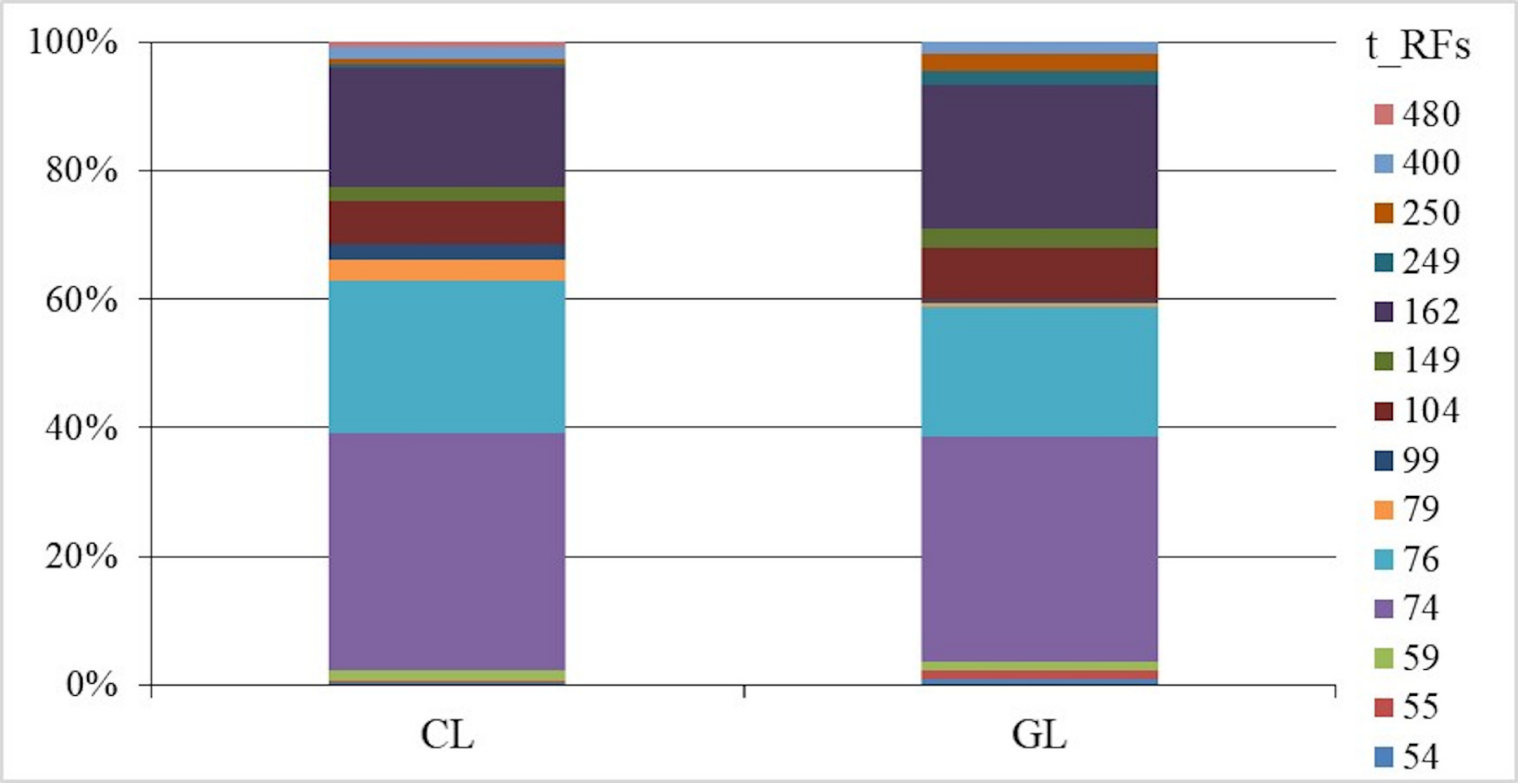
Relative abundance (%) of ammonia-oxidizing archaea (AOA) amoA gene sequences fragments (T-RFs) after AluI digestion in cropland (CL) and grassland (GL) soils. T_RFs, Restriction fragments with different size. Fragments size is expressed as base pairs (bp) from 54 to 480 bp.

The t-RFLP analysis showed moderate changes in the microbial community of ammonia-oxidizing archaea between cropland and grassland soils (Fig. 5). The diversity of the archaeal ammonia oxidizers based on the *amo*A gene revealed 14 different terminal restriction fragments detected in both tested soil type uses (cropland and grassland). The peaks ranged from 54 to 480 bp and were obtained after restriction with AluI enzyme. The distribution in relative abundance of the various fragment sizes suggests that there are different t-RFLP diversity patterns between the tested cropland and grassland soils. The most intensive t-RFs fragments present in both types of soil were the following: 74, 76, 104 and 162 bp. A higher intensity of fragments with lower sizes: 74, 76, 79 and 99 bp were found in cropland rather than in grassland soils, while larger fragment sizes with 104, 162 and 250 bp were more intensive in grassland compared to cropland soils (Fig. 5). In silico analysis using the amoA_AOA Feifei-Liu reference database of FunGene (Fish et al., 2013) revealed that the most common restriction fragment t-RF 162 was assigned to different uncultured ammonia-oxidizing archaeon members of Thaumarchaeota, which may include *Candidatus* Nitrososphaera viennensis, *Candidatus* Nitrososphaera evergladensis and *Candidatus* Nitrosoarchaeum limnia. Moreover, based on in silico analysis using TRiFLe software (Junier, Junier & Witzel, 2008), the higher t-RFs obtained could be assigned to the *Nitrosopumilus* genus including *N. cobalaminigenes*, *N. oxyclinae*, *N. ureiphilus* and *Candidatus* Nitrosotenuis, while lower fragments could be associated with *Candidatus* Nitrosotalea.

### Fungal community structure of soils

In total, 45,125 high-quality sequences from both tested soil types were obtained. The sequences were classified into fungal sequences by UNITE databases. In precise terms, we obtained 23,901 and 21,224 sequences for cropland and grassland soils, respectively (Table 1).

**Table 1:** Mean values of microbiological and physical indicators of cropland (CL) and grassland (GL) soils.

Soil indicator	Cropland (CL)	Grassland (GL)
BR (mg CO_2_–C kg^−1^ h^−1^)	3.85 ± 0.44	7.31 ± 3.38
MBC (mg C g^−1^)	0.36 ± 0.14	1.12 ± 0.69
DA (mg TPF kg^−1^ d^−1^)	13.46 ± 5.03	49.56 ± 37.46
BA (mg PNP kg^−1^ h^−1^)	197.54 ± 72.94	371.37 ± 267.03
pH	5.49 ± 0.53	5.22 ± 0.43
BD (Mg m^−3^)	1.24 ± 0.03	1.28 ± 0.07
WHC (m^3^ m^−3^)	0.18 ± 0.03	0.27 ± 0.07
SOC (%)	1.06 ± 0.29	1.76 ± 0.32
Total number of reads	23,901	21,224
Richness (OD_490_ > 0.25)	15.44 ± 6.63	17.81 ± 3.96

**Note:**

BR, Basal respiration; MBC, Microbial biomass; DA, Dehydrogenase activity; BA, β-glucosidase activity; BD, Bulk density; WHC, Water holding capacity; SOC, Soil organic carbon.

**Figure 6:**
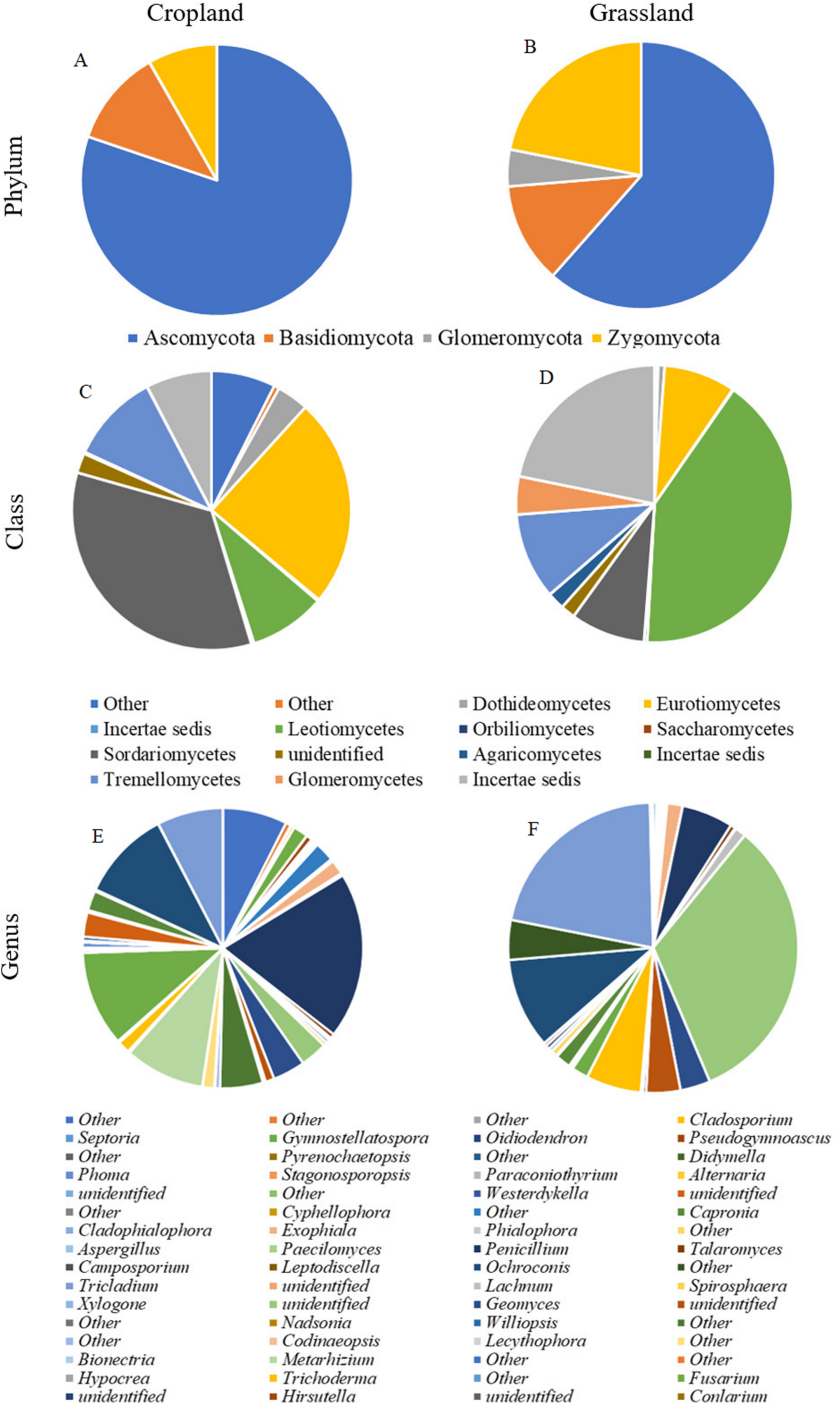
Sunburst plot showing the taxonomic relative abundance of sequences to phylum, class and genus level of fungi for cropland (CL) and grassland (GL) soils. (A) Taxonomic relative abundance at the phylum level in cropland, (B) taxonomic relative abundance at the phylum level in grassland, (C) taxonomic relative abundance at the class level in cropland, (D) taxonomic relative abundance at the class level in grassland, (E) taxonomic relative abundance at the genus level in cropland, (F) taxonomic relative abundance at the genus level in grassland.

For both of the tested soil types (cropland and grassland) we found only two phyla: Ascomycota and Basidiomycota with relative abundances ranging from 74% to 11% for cropland and 61–12% for grassland, respectively. In addition, in cropland Incertae sedis or other unclassified fungal sequences were at the level of 8% and 7%, respectively. In grassland, 22% and 4% of the total number of sequences were Incertae sedis and other sequences unassigned to any fungal phyla, respectively (Figs. 6A and 6B; Figs. S1 and S2).

The results indicated evident differences between cropland and grassland at the class level of taxonomic classification (Figs. 6C and 6D; Figs. S1 and S2). On the basis of the total high-quality sequences, Sordariomycetes and Eurotiomycetes were the two dominant classes in cropland, accounting for 34% of Fungi and 46% of Ascomycota and 24% of Fungi and 33% of Ascomycota, respectively. Furthermore, two other classes of fungi: Leotiomycetes and Dothiomycetes, which accounted for an average of 9% and 4% of the total sequences, respectively were detected in the present study in cropland. These classes (Leotiomycetes and Dothiomycetes) in cropland were at the level of 12% and 5% of the Ascomycota phylum, respectively. In grassland, Leotiomycetes and Mortierella were the two dominant classes accounting for 41% of Fungi and 67% of Ascomycota, and 22% of Fungi and 99% of Incertae sedis, respectively. Sordariomycetes and Eurotiomycetes constituted only 9% of Fungi and 14% of Ascomycota and 8% of Fungi and 14% of Ascomycota, respectively.

Further taxonomical classification at the genus level revealed that the fungal community in cropland was dominated by *Penicillium*, *Fusarium*, *Metarhizium* and *Mortierella* (Figs. 6E and 6F; Fig. S1) which account for 19%, 11%, 9% and 8% of the total sequences, respectively. The most dominant fungal genera in grassland were the following: *Helotiales* (33%), *Mortierella* (22%), *Tremellales* (10%) and *Trichoderma* (6%). However, in cropland, representatives of the *Trichoderma* genus were at a lower level—about 1% of the total counts of Fungi.

To further illustrate the differences in the composition of the fungal community, it is worth mentioning that the most frequently detected species in cropland was *Metarhizium anisopliae* (9% of Fungi, 12% of Ascomycota) (Fig. S1) while in grassland it was *Mortierella globulifera* (7% of Fungi, 33% of Mortierella) (Fig. S2).

**Figure 7:**
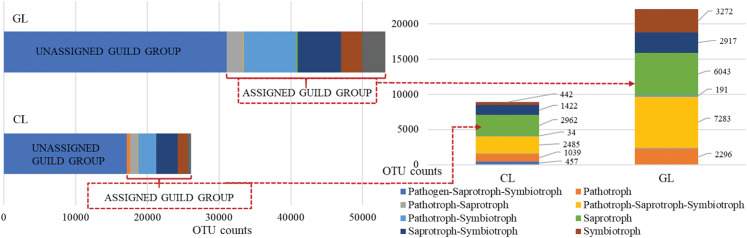
Guild assignments for cropland (CL) and grassland (GL) soils based on OTU richness assigned to fungal trophic guilds.

Taking into account the ecological guilds in the fungal community we observed that OUT richness varied between both of the tested treatments of cropland and grassland (Fig. 7). It was found that unassigned groups dominated both cropland and grassland soils, constituting 58% and 44% of the total, respectively (Figs. S3 and S4). When unassigned OTUs were removed, saprotrophs were found to be the largest guild in cropland, with counts of 2,962 OTUs (Fig. 7). The second largest group of guilds included the pathotroph–saprotroph–symbiotroph group (2,485 OTUs) with fungal parasites and unidentified saprotrophs (36%) dominating, animal pathogen, dung saprotroph, endophyte, epiphyte, plant saprotroph and wood saprotroph (22%) and animal pathogen, endophyte, lichen parasite, plant pathogen, soil and wood saprotroph (22%), these results are presented in Fig. S3. The opposite situation was observed in grassland soil where pathotroph–saprotroph–symbiotroph (7,283 OTUs) were the largest guild with fungal parasites and unidentified saprotrophs (62%) dominating, and the second one was saprotrophs (6,043 OTUs) with the domination of undefined saprotrophs (73%), soil saprotrophs (20%) and wood saprotrophs (6%), which were depicted in Fig. S4.

## Discussion

### Effects of land use type on soil microbial and enzymatic activity

Microbial biomass content and basal respiration rate are considered to be sensitive indicators of changes in soil organic matter quality (Sparling, 1992). Cropland Podzol soil showed significantly lower values in both parameters compared to grassland soil, indicating better organic matter quality in grassland soil, this is probably connected with the higher accumulation of organic substrates and the extensive root system of grass, which creates better conditions for the growth of microbial biomass (Dilly et al., 2001; Maková et al., 2011).

Soil enzymatic activity is very important for soil biochemical functioning. Therefore, it may be a useful biomarker for monitoring the influence of land use type on the state of the soil (Acosta-Martinez et al., 2007). PCA and correlation analyses have shown the significant separation of enzymatic activity between the tested land use types. The results also demonstrated a significant decline in dehydrogenase and β-glucosidase activities in cropland. Similar trends in the comparison of bare fallow and grassland were described by Kot et al. (2015). In our study, we found a significant relationship between SOC, WHC and DA in both lands uses and between SOC and BA in grassland soil (Fig. 1). Higher β-glucosidase activities in grassland soil may be connected with high grass root turnover or the absence of tillage (Acosta-Martinez et al., 2007). The results obtained agree with the other relevant studies (Bandick & Dick, 1999) indicating a higher enzymatic activity in grassland soil because of the extensive accumulation of biomass including cellulosic grass roots as compared to cropland soil. The differences in enzymatic activity, especially in DA which are observed between plots I, II and III were also connected with soil moisture. Therefore, the highest dehydrogenases activity was noted in soil with a higher water content (26%). The higher relative abundance of some individual terminal restriction fragments of AOA in cropland soils indicates changes in the community structure of these microorganisms and suggests more intensive nitrification rates than in grassland. This may be explained by the production of specific root exudates by grassland plants, which may inhibit nitrification (Norton & Ouyang, 2019). Moreover, the rhizosphere microbes of grassland can produce a wide array of signalling molecules (Ladygina, Dedyukhina & Vainshtein, 2006) that may cause nitrification inhibitory effects (Norton & Ouyang, 2019). Additionally, changes in the relative abundance of ammonia-oxidizing archaea in cropland and grassland can also be explained by the soil aggregate level. It has been established that in cropland soil the small macroaggregates provide the necessary microenvironment for AOA growth, thereby producing potential hotspots for ammonia oxidation (Chen et al., 2016). The predominant t-RF 162 in both cropland and grassland soils could be explained by the features of different members of *Candidatus* Nitrososphaera, that are capable of biofilm formation, detoxification and adhesion, thus they are well adapted to environmental changes (Hammerl et al., 2019; Kerou et al., 2016). Moreover, the members of Thaumarchaeota, for example several species classified in the *Nitrosotalea* genus, may be adapted to acidic environments with a pH lower than 6.5 (Ginawi et al., 2020), therefore in our study their representatives were found in both tested sandy acidic soils under cropland and grassland. It should be taken into consideration that the assignment of archaeal community was based on databases in silico analysis. However, the presence of *Candidatus* Nitrososphaera, *Candidatus* Nitrosoarchaeum limnia, *Nitrosopumilus* sp., *Candidatus* Nitrosotenuis and *Candidatus* Nitrosotalea were detected in different sediment and soil environments (Hammerl et al., 2019; Cao et al., 2012; Ginawi et al., 2020; Wessén et al., 2011).

### Effects of land use type on the soil fungal community

The land use type significantly influences biodiversity causing changes to the relative abundance and structure of microbial communities (Wang et al., 2017; Thomson et al., 2015; Szoboszlay et al., 2017). Our results have shown that conversion to grassland led to an increase in fungal richness and diversity compared to cropland. A number of studies have confirmed that soil microbes are often changed by tillage, management practices and land use (Yao et al., 2017; Wang et al., 2016). Different ecosystems and land uses have various phytosanitary capacities, which refers to their ability to protect plants against pests and diseases and is also associated with the presence of beneficial bacteria, fungi and other mechanisms supporting biosecurity and protecting biodiversity (Food and Agriculture Organization of the United Nations, 2017). Bacterial communities are rather well characterized in cropland and grassland ecosystems (Kaiser et al., 2016; Kot et al., 2015), however, little is known about the fungal community compositions of sandy acidic soils under cropland and grassland.

In the present study, the results showed that different land use types had a significant effect on the soil fungal community. It was found that Sordariomycetes and Eurotiomycetes were the most dominant fungal classes in cropland, whereas Leotiomycetes and Mortierella dominated in grassland. Sordariomycetes consists of fungi, which are very often detected in agricultural soils (Ding et al., 2017). What is more, these fungi increase in number in nitrogen-rich soils (Mueller, Belnap & Kuske, 2015) and are able to decompose organic residues incorporated into the soil (Strope et al., 2011). This information may explain the higher abundance of Sordariomycetes in cropland as opposed to grassland soils. Fungi belonging to the Eurotiomycetes class are resistant to stress conditions because they can adapt to them (e.g., acidity, salinity, heat, drought) they are also capable of living in extreme ecosystems (Nai et al., 2013; Sterflinger, Tesei & Zakharova, 2012). Moreover, these fungi demonstrate cellulolytic abilities by producing cellulases, which participate in carbon compound decomposition (Fan et al., 2012). The results of our study are based on ecological guilds obtained by FUNGuild® for cropland soils and indicated that a huge percentage of the fungal community participated in wood decomposition, which confirmed enzyme secretion by these fungi. Therefore, the above-mentioned abilities may facilitate their presence in arable acidic sandy soils. What is more, due to their metabolic functions in carbon turnover and energy flow, both Sordariomycetes and Eurotiomycetes participate in the biogeochemical transformations of SOC (Chen et al., 2012, 2015).

In comparison, the relative abundances of Sordariomycetes and Eurotiomycetes were significantly lower in grassland than in cropland soils. Representatives of Eurotiomycetes are widely distributed as denitrification drivers (Mueller, Belnap & Kuske, 2015) causing losses in nitrous oxide from soil and atmospheric pollution, and therefore a lower abundance of these microbes in grassland indicates the positive influence on soil quality and environmental protection. Greater abundances of Mortierella and Leotiomycetes with the domination of Helotiales were found under grassland. Members of the genera Mortierella were found in arable soils (Liu et al., 2015), woodland soils (Franke-Whittle et al., 2015) and soils with cover crops cultivation (Detheridge et al., 2016). The abundance of Mortierella was positively correlated with soil nitrate-N and negatively with soil P (Detheridge et al., 2016). Additionally, according to Zhang et al. (2014), Mortierella may play a role in the transformation of inorganic sources of P by the secretion of organic acids. Nallanchakravarthula et al. (2014) suggest that Mortierella is among the endophytic, mycorrhizal fungi, which are most abundant in the soil rhizosphere. An analysis of ecological guilds over the course of our research confirmed that endophytic and mycorrhizal fungi dominated in grassland soils. All of these findings can be used to explain the relatively high abundance of Mortierella in grassland soils with a potentially high content of nitrate and a low content of phosphorus as well as an extensive rhizosphere system created by the roots of grasses. The high abundance of Leotiomycetes in grassland soils can be explained by the significant abilities of these fungi in the area of lignocellulose degradation (Yoon et al., 2010; Sterflinger, Tesei & Zakharova, 2012) due to the high content of substrates, which can stimulate their growth in grassland soils, that is, rich-lignocellulose grass residues and an extensive root system in grassland soils. Dominant Helotiales have been found previously in arctic soils and appear more frequently in warm conditions (Deslippe et al., 2012) and agricultural soils (Klaubauf et al., 2010), they are known as ectomycorrhizal fungi (Deslippe et al., 2012).

It is worth noting that the grassland soils were found to be richer in antagonistic, beneficial fungi from the genus *Trichoderma*, suggesting the natural properties of grassland in disease suppression through the presence of disease-suppressing fungi. Taking into consideration the results of ecological guild groups, it was found that fungal parasites, endophytes, ectomycorrhizal and endomycorrhizal fungi were present, and that they were dominant, especially in grassland soil, thereby confirming its natural suppressiveness to pathogens, which may be defined as the capacity of the soil to regulate soil-borne pathogens (Bongiorno et al., 2019).

### Overall interactions of soil quality

In this study, we observe the correspondence between the microbiological and physical parameters of the soil, although the correlations between them were dependent on the type of land use. Therefore, different soil properties should be taken into consideration during the evaluation of soil quality under different land uses. Soil organic carbon correlates with many soil properties, therefore they may be used for soil quality monitoring (Delelegn et al., 2017). The results of our study demonstrated that SOC was associated with water holding capacity (WHC), soil enzymes (DA, BA) and microbial biomass (MBC). In this study, we observed that cropland soils had a lower SOC, BD and WHC and that this was linked to a reduction in microbial biomass and to a relative abundance of fungi.

On the basis of the study, we recommend that the soil fungal community composition should be treated as a very important indicator of soil quality evaluation of cropland and grassland. This indicator informs us not only about the relative abundance of fungi but also, due to the fact that there are many new sequence databases, it can be used for the evaluation of the functions of fungi present in the soil environment and to assess the presence of pathogenic and antagonistic fungi in soils. This may be useful in agriculture management for high-level productivity. Nevertheless, the methodological aspects of next generation sequencing should be improved in order to identify the unassigned fungi present in the soil environment.

## Conclusions

Our study showed that grassland (present for 25 years) compared to cropland had a significantly higher organic carbon content, microbial biomass, basal respiration, enzyme activities, fungal diversity, richness and diversity of the soil fungal community and water holding capacity. The principal component analysis (PCA) of soil properties indicated the general differences between land use type (grassland and cropland). MBC, WHC, DA, BA, pH, SOC in grassland and MBC and DA in cropland had the most influence over soil variability.

Conversion to grassland increased soil fungal and ammonia-oxidizing archaeal relative abundances and changed the structure of fungal communities. Moreover, we observed a significant decrease in the relative abundance of potential crop pathogens (especially *Penicillium* sp. and *Fusarium* sp.) in grassland and an increase in *Trichoderma* sp. (beneficial and antagonistic fungus) compared to cropland. It is worth emphasizing that the results suggest that grassland use can have a phytosanitary capacity in ecosystem functioning, this was observed in the decrease in the number of fungal pathogens in grassland compared to cropland soils. Although changes in the relative abundance of AOA were observed between cropland and grassland soils, in silico analysis indicated that uncultured ammonia-oxidizing archaea of Thaumarchaeota, such as *Candidatus* Nitrososphaera viennensis, *Candidatus* Nitrososphaera evergladensis and *Candidatus* Nitrosoarchaeum limnia, *Nitrosopumilus* sp., *Candidatus* Nitrosotenuis and Candidatus *Nitrosotalea* were found in both of the tested sandy acidic soils. Moreover, the results suggested a status improvement in soil organic carbon and nitrogen dynamics by increasing the relative abundance of fungi and ammonia-oxidizing archaea.

All of the results obtained will contribute to an improved understanding of the differences between cropland and grassland and the link between soil quality and the soil microbial community, especially fungal diversity, as significant, qualitative and quantitative components of complex microbial communities.

## Supplemental Information

10.7717/peerj.9501/supp-1 Supplemental Information 1 Krona chart of the fungi represented by ITS1 region sequences recovered from cropland.

10.7717/peerj.9501/supp-2 Supplemental Information 2 Krona chart of the fungi represented by ITS1 region sequences recovered from grassland.

10.7717/peerj.9501/supp-3 Supplemental Information 3 Krona chart with ecological guild assignments for fungal sequences dataset from cropland using FUNGuild.

10.7717/peerj.9501/supp-4 Supplemental Information 4 Krona chart with ecological guild assignments for fungal sequences dataset from grassland using FUNGuild.

10.7717/peerj.9501/supp-5 Supplemental Information 5 Raw data.

## Additional Information and Declarations

### Field Study Permissions

The following information was supplied relating to field study approvals (i.e., approving body and any reference numbers): The access authorization to the fields were given verbally by farmer Marek Lasocki, owner of fields included into our manuscript and the Agricultural Farm in Trzebieszów, Poland.

### Data Availability

The following information was supplied regarding data availability: The Illumina sequencing data are available at NCBI SRA: SRP131723 and the raw data are also available in the Supplemental Files.
