# Supplementary material for: Structural and functional microbial diversity of sandy soil under cropland and grassland
Source: PeerJ. 2020 Sep 2;8:e9501. doi: 10.7717/peerj.9501 (PMC7474522; doi:10.7717/peerj.9501)
Supplement: Supplemental Information 1 [file peerj-08-9501-s001.html]

Javascript must be enabled to view this page.

magnitude
 6116
 6116
 454
 454
 454
 454
 454
 454
 4542
 37
 37
 37
 37
 37
 227
 29
 7
 7
 7
 22
 22
 22
 0
 0
 0
 146
 105
 104
 104
 1
 1
 41
 41
 41
 52
 1
 1
 1
 8
 8
 8
 17
 3
 3
 5
 5
 9
 9
 0
 0
 9
 9
 9
 5
 3
 3
 2
 2
 11
 11
 11
 0
 0
 1
 1
 1
 1487
 263
 0
 0
 0
 2
 2
 2
 261
 140
 140
 0
 0
 18
 18
 103
 79
 17
 7
 1224
 13
 13
 13
 1211
 3
 3
 1
 1
 1175
 1175
 0
 32
 32
 12
 12
 12
 1
 1
 1
 1
 10
 10
 540
 249
 7
 7
 7
 25
 25
 25
 1
 1
 1
 26
 25
 25
 1
 1
 190
 190
 9
 181
 229
 229
 229
 208
 21
 62
 62
 62
 62
 12
 12
 12
 12
 12
 8
 8
 3
 3
 3
 5
 5
 5
 2078
 302
 302
 302
 302
 39
 39
 34
 34
 5
 5
 1
 1
 1
 1
 1466
 82
 82
 82
 3
 3
 3
 565
 565
 565
 0
 0
 0
 99
 5
 5
 8
 8
 86
 40
 4
 42
 688
 14
 14
 673
 673
 1
 1
 18
 18
 0
 18
 11
 11
 11
 43
 3
 3
 3
 2
 2
 2
 1
 1
 1
 37
 37
 37
 0
 0
 0
 0
 2
 2
 1
 1
 1
 1
 8
 8
 8
 8
 210
 2
 2
 2
 200
 28
 28
 172
 172
 0
 0
 0
 8
 8
 8
 7
 7
 7
 7
 141
 141
 141
 141
 131
 10
 652
 11
 2
 0
 0
 0
 1
 1
 1
 1
 1
 1
 0
 0
 0
 0
 0
 0
 3
 3
 3
 3
 0
 0
 0
 0
 6
 0
 0
 0
 6
 6
 6
 1
 1
 1
 1
 1
 640
 640
 640
 640
 640
 2
 2
 2
 2
 2
 2
 466
 466
 464
 464
 464
 419
 2
 0
 5
 36
 2
 2
 2
 2
 2
 0
 0
 0
 0
 0
 0
