# Supplementary material for: Structural and functional microbial diversity of sandy soil under cropland and grassland
Source: PeerJ. 2020 Sep 2;8:e9501. doi: 10.7717/peerj.9501 (PMC7474522; doi:10.7717/peerj.9501)
Supplement: Supplemental Information 2 [file peerj-08-9501-s002.html]

Javascript must be enabled to view this page.

magnitude
 7577
 7577
 26
 26
 26
 26
 26
 26
 4638
 6
 6
 6
 6
 6
 57
 6
 2
 2
 2
 3
 3
 3
 1
 1
 1
 28
 27
 20
 20
 7
 7
 1
 1
 1
 23
 0
 0
 0
 10
 10
 10
 3
 0
 0
 0
 0
 0
 0
 3
 3
 0
 0
 0
 0
 0
 0
 0
 0
 10
 0
 0
 10
 10
 0
 0
 0
 635
 159
 4
 4
 4
 1
 1
 1
 154
 20
 20
 2
 2
 1
 1
 131
 130
 0
 1
 476
 0
 0
 0
 476
 0
 0
 1
 1
 433
 417
 16
 42
 42
 7
 7
 7
 0
 0
 6
 6
 1
 1
 3117
 2575
 0
 0
 0
 1
 1
 1
 93
 93
 93
 0
 0
 0
 0
 0
 2481
 2481
 2
 2479
 254
 254
 254
 238
 16
 288
 288
 288
 288
 28
 28
 28
 28
 28
 0
 0
 0
 0
 0
 0
 0
 0
 660
 3
 3
 3
 3
 0
 0
 0
 0
 0
 0
 1
 1
 1
 1
 645
 3
 3
 3
 1
 1
 1
 0
 0
 0
 9
 9
 9
 467
 0
 0
 0
 0
 467
 456
 11
 0
 140
 0
 0
 140
 140
 0
 0
 14
 14
 13
 1
 11
 11
 11
 0
 0
 0
 0
 0
 0
 0
 0
 0
 0
 0
 0
 0
 1
 1
 1
 1
 4
 4
 0
 0
 4
 4
 1
 1
 1
 1
 5
 0
 0
 0
 1
 0
 0
 1
 1
 4
 4
 4
 0
 0
 0
 0
 0
 0
 0
 128
 128
 128
 128
 114
 14
 917
 153
 10
 10
 10
 10
 0
 0
 0
 0
 0
 0
 83
 83
 51
 51
 32
 32
 0
 0
 0
 0
 33
 33
 33
 33
 27
 27
 27
 27
 0
 0
 0
 0
 0
 0
 0
 0
 764
 764
 764
 764
 764
 336
 336
 336
 336
 336
 336
 1649
 1649
 1633
 1633
 1633
 972
 32
 2
 545
 0
 82
 16
 16
 16
 16
 11
 11
 11
 11
 11
 11
