# Supplementary material for: Structural and functional microbial diversity of sandy soil under cropland and grassland
Source: PeerJ. 2020 Sep 2;8:e9501. doi: 10.7717/peerj.9501 (PMC7474522; doi:10.7717/peerj.9501)
Supplement: Supplemental Information 3 [file peerj-08-9501-s003.html]

Javascript must be enabled to view this page.

magnitude
 21363
 12443
 12443
 1039
 220
 775
 40
 4
 79
 1
 5
 1
 1
 10
 14
 6
 3
 38
 2485
 1
 7
 2
 558
 907
 198
 548
 143
 2
 22
 2
 5
 11
 6
 66
 7
 34
 6
 1
 15
 12
 2962
 2019
 288
 135
 297
 29
 17
 2
 29
 3
 33
 4
 8
 98
 1422
 40
 1259
 1
 35
 14
 15
 49
 9
 442
 74
 20
 39
 184
 125
 457
 457
