# Supplementary material for: Structural and functional microbial diversity of sandy soil under cropland and grassland
Source: PeerJ. 2020 Sep 2;8:e9501. doi: 10.7717/peerj.9501 (PMC7474522; doi:10.7717/peerj.9501)
Supplement: Supplemental Information 4 [file peerj-08-9501-s004.html]

Javascript must be enabled to view this page.

magnitude
 39534
 17429
 17429
 103
 1
 36
 58
 8
 7283
 1
 4507
 426
 95
 888
 183
 45
 2
 34
 489
 13
 520
 80
 6043
 4409
 1
 1218
 357
 23
 35
 3272
 1619
 260
 1234
 159
 2917
 2165
 57
 361
 261
 73
 2296
 1058
 1226
 4
 8
 191
 191
